# Molecular epidemiology of SARS-CoV-2 isolated from COVID-19 family clusters

**DOI:** 10.1186/s12920-021-00990-3

**Published:** 2021-06-01

**Authors:** Gunadi, Hendra Wibawa, Mohamad Saifudin Hakim, Marcellus, Ika Trisnawati, Riat El Khair, Rina Triasih, Irene, Afiahayati, Kristy Iskandar, Siswanto, Nungki Anggorowati, Edwin Widyanto Daniwijaya, Endah Supriyati, Dwi Aris Agung Nugrahaningsih, Eko Budiono, Heni Retnowulan, Yunika Puspadewi, Ira Puspitawati, Osman Sianipar, Dwiki Afandy, Susan Simanjaya, William Widitjiarso, Dyah Ayu Puspitarani, Fadil Fahri, Untung Riawan, Aditya Rifqi Fauzi, Alvin Santoso Kalim, Nur Rahmi Ananda, Amalia Setyati, Dwikisworo Setyowireni, Ida Safitri Laksanawati, Eggi Arguni, Titik Nuryastuti, Tri Wibawa, the Yogyakarta-Central Java COVID-19 study group, Elisabeth S. Herini, Titis Widowati, Cahya Dewi Satria, Sumardi, Bambang Sigit Riyanto, Munawar Gani, Satria Maulana, Ludhang Pradipta Rizki, Umi Solekhah Intansari, ‬Elizabeth Henny Herningtiyas, Nur Imma Fatimah Harahap, Bagoes Poermadjaja, Sintong H. M. T. Hutasoit, Indaryati, Havid Setyawan, Kemala Athollah, Maria Patricia Inggriani

**Affiliations:** 1 Pediatric Surgery Division, Department of Surgery/Genetics Working Group, Faculty of Medicine, Public Health and Nursing, Universitas Gadjah Mada/Dr, Sardjito Hospital, Jl. Kesehatan No. 1, Yogyakarta, 55281 Indonesia; 2 Disease Investigation Center Wates, Directorate General of Livestock and Animal Health Services, Ministry of Agriculture, Yogyakarta, Indonesia; 3 Department of Microbiology, Faculty of Medicine, Public Health and Nursing, Universitas Gadjah Mada, Yogyakarta, Indonesia; 4 Genetics Working Group, Faculty of Medicine, Public Health and Nursing, Universitas Gadjah Mada, Yogyakarta, Indonesia; 5 Pulmonology Division, Department of Internal Medicine, Faculty of Medicine, Public Health and Nursing, Universitas Gadjah Mada/Dr, Sardjito Hospital, Yogyakarta, Indonesia; 6 Department of Clinical Pathology and Laboratory Medicine, Faculty of Medicine, Public Health and Nursing, Universitas Gadjah Mada/Dr, Sardjito Hospital, Yogyakarta, 55281 Indonesia; 7 Department of Child Health, Faculty of Medicine, Public Health and Nursing, Universitas Gadjah Mada/Dr, Sardjito Hospital, Yogyakarta, Indonesia; 8 Balai Besar Teknik Kesehatan Lingkungan Dan Pengendalian Penyakit, Yogyakarta, Yogyakarta, Indonesia; 9 Department of Computer Science and Electronics Faculty of Mathematics and Natural Sciences, Universitas Gadjah Mada, Yogyakarta, Indonesia; 10 Department of Child Health/Genetics Working Group, Faculty of Medicine, Public Health and Nursing, Universitas Gadjah Mada/UGM Academic Hospital, Yogyakarta, Indonesia; 11 Department of Physiology, Faculty of Medicine, Public Health and Nursing, Universitas Gadjah Mada/UGM Academic Hospital, Yogyakarta, Indonesia; 12 Department of Anatomical Pathology/Genetics Working Group, Faculty of Medicine, Public Health and Nursing, Universitas Gadjah Mada, Yogyakarta, Indonesia; 13 Department of Microbiology, Faculty of Medicine, Public Health and Nursing, Universitas Gadjah Mada, UGM Academic Hospital, Yogyakarta, Indonesia; 14 Centre of Tropical Medicine, Faculty of Medicine, Public Health and Nursing, Universitas Gadjah Mada, Yogyakarta, Indonesia; 15 Department of Pharmacology and Therapy/Genetics Working Group, Faculty of Medicine, Public Health and Nursing, Universitas Gadjah Mada, Yogyakarta, Indonesia

**Keywords:** COVID-19 severity, Family cluster, Multiple spike protein mutations, Phylogenetic analysis, SARS-CoV-2 transmission, Whole genome sequencing

## Abstract

**Background:**

Transmission within families and multiple spike protein mutations have been associated with the rapid transmission of SARS-CoV-2. We aimed to: (1) describe full genome characterization of SARS-CoV-2 and correlate the sequences with epidemiological data within family clusters, and (2) conduct phylogenetic analysis of all samples from Yogyakarta and Central Java, Indonesia and other countries.

**Methods:**

The study involved 17 patients with COVID-19, including two family clusters. We determined the full-genome sequences of SARS-CoV-2 using the Illumina MiSeq next-generation sequencer. Phylogenetic analysis was performed using a dataset of 142 full-genomes of SARS-CoV-2 from different regions.

**Results:**

Ninety-four SNPs were detected throughout the open reading frame (ORF) of SARS-CoV-2 samples with 58% (54/94) of the nucleic acid changes resulting in amino acid mutations. About 94% (16/17) of the virus samples showed D614G on spike protein and 56% of these (9/16) showed other various amino acid mutations on this protein, including L5F, V83L, V213A, W258R, Q677H, and N811I. The virus samples from family cluster-1 (n = 3) belong to the same clade GH, in which two were collected from deceased patients, and the other from the survived patient. All samples from this family cluster revealed a combination of spike protein mutations of D614G and V213A. Virus samples from family cluster-2 (n = 3) also belonged to the clade GH and showed other spike protein mutations of L5F alongside the D614G mutation.

**Conclusions:**

Our study is the first comprehensive report associating the full-genome sequences of SARS-CoV-2 with the epidemiological data within family clusters. Phylogenetic analysis revealed that the three viruses from family cluster-1 formed a monophyletic group, whereas viruses from family cluster-2 formed a polyphyletic group indicating there is the possibility of different sources of infection. This study highlights how the same spike protein mutations among members of the same family might show different disease outcomes.

**Supplementary Information:**

The online version contains supplementary material available at 10.1186/s12920-021-00990-3.

## Introduction

Many countries are still struggling to control the COVID-19 pandemic, including Indonesia [1, 2]. On April 15, 2021, Indonesia recorded 1,583,182 confirmed COVID-19 cases with 42,906 deaths and infection rate of approximately 6000 cases/day [3].

One of the most important factors affecting the rapid spreading of COVID-19 is transmission within families [4, 5]. Genomic epidemiology has been suggested to be important to fill the gaps in identifying the SARS-CoV-2 infection sources [6]. However, to our best knowledge, no reports have described the genomic epidemiology within family clusters [6–8]. Moreover, multiple spike protein mutations have been associated with a higher transmissibility of SARS-CoV-2 [9]. In this study, we aimed to: (1) perform full genome characterization of SARS-CoV-2 and correlate the sequences with the epidemiological data within family clusters in Indonesia, and (2) conduct phylogenetic analysis of all samples from Yogyakarta and Central Java, Indonesia, involving the family clusters, and virus data from other regions in Indonesia.

## Methods

### SARS-CoV-2 samples

We collected all virus samples of confirmed COVID-19 patients from Yogyakarta and Central Java provinces from June to November 2020. All nasopharyngeal samples were collected in viral transport media (DNA/RNA Shield™ Collection Tube with Swab, Zymo Research, CA, United States) and transported to four COVID-19 diagnostic laboratories in Yogyakarta province: (1) Molecular Diagnostic Laboratory, Integrated Laboratory Unit, Dr. Sardjito Hospital; (2) Department of Microbiology and Laboratorium Diagnostik Yayasan Tahija World Mosquito Program, Faculty of Medicine, Public Health and Nursing, Universitas Gadjah Mada; (3) Balai Besar Teknik Kesehatan Lingkungan dan Pengendalian Penyakit (BBTKLPP), Yogyakarta; and (4) Disease Investigation Center, Wates, Yogyakarta. SARS-CoV-2 was detected by Real-Q 2019-nCoV Detection Kit (BioSewoom, Seoul, South Korea) with LightCycler® 480 Instrument II (Roche Diagnostics, Mannheim, Germany).

### Full-genome sequencing

First, we performed RNA extraction of 19 nasopharyngeal swab samples by a QiAMP Viral RNA mini kit (Qiagen, Hilden, Germany), synthesized the double-stranded cDNA by Maxima H Minus Double-Stranded cDNA Synthesis (Thermo Fisher Scientific, MA, United States), and purified the cDNA using a GeneJET PCR Purification Kit (Thermo Fisher Scientific, MA, United States). For library preparations, we utilized the Nextera DNA Flex for Enrichment using Respiratory Virus Oligos Panel, whereas for full-genome sequencing, we used next generation sequencing (NGS) applied in the Illumina MiSeq instrument (Illumina, San Diego, CA, United States) with Illumina MiSeq reagents v3 150 cycles (2 × 75 cycles). We excluded two samples for further bioinformatics analysis because of low coverages. Our sample genomes were assembled by mapping to the reference genome from Wuhan, China (hCoV-19/Wuhan/Hu-1/2019, GenBank accession number: NC_045512.2) using Burrow-Wheeler Aligner (BWA) algorithm embedded in UGENE v. 1.30 [10]. Identification of single nucleotide polymorphisms (SNPs) was performed using the number of high confidence base calls (consensus sequence variations of the assembly) that disagree with the reference bases for the genome position of interest, then all SNPs were exported to a vcf. file and visualized in MS Excel. The following accession IDs for the 17 samples are: EPI_ISL_516800, EPI_ISL_516806, EPI_ISL_516829, EPI_ISL_525492, EPI_ISL_576383, EPI_ISL_632936, EPI_ISL_610161, EPI_ISL_610162, EPI_ISL_576145, EPI_ISL_632937, EPI_ISL_575331, EPI_ISL_576113, EPI_ISL_576114, EPI_ISL_576115, EPI_ISL_576116, EPI_ISL_576128, and EPI_ISL_576130 [11]. The first four IDs have been reported in our previous study [12].

### Phylogenetic analysis

We used the reference genome of hCoV-19/Wuhan/Hu-1/2019 (NC_045512.2) for annotation of our sequences. A dataset of 142 available SARS-CoV-2 genomes (89 sequences from Indonesia and 53 from other countries) was retrieved from GISAID to conduct a phylogenetic analysis (Acknowledgment Table is provided in Additional file 2: Table S2). We only used the full-genome sequences of several strains representing SARS-CoV-2 clades from some countries that had complete genome data and no long stretches of ‘NNNN’ for the phylogenetic analysis. The MAFFT program server was utilized for multiple nucleotide sequence alignment (https://mafft.cbrc.jp/alignment/server/). A phylogenetic tree was constructed from 29.409 nt length of the open reading frame (ORF) of 142 SARS-CoV-2 virus sequences using Neighbor Joining statistical method with 2000 bootstrap replications. The evolutionary distances were computed using the Kimura 2-parameter method and the rate variation among sites was modelled using a gamma distribution with estimated shape parameter (α) for the dataset. The estimation of α gamma distribution was calculated in DAMBE version 7 [13], whereas all the other analyses were performed in MEGA version 10 (MEGA X) [14].

### COVID-19 severity classifications

COVID-19 severity was determined based on the WHO classifications: (1) mild, without evidence of hypoxia or pneumonia; (2) moderate, pneumonia but not severe; (3) severe, pneumonia plus one of the following signs: respiratory rate > 30 breaths/minute (or based on age for children), severe respiratory distress, or SpO_2_ < 90% in room air; and (4) critical, Acute Respiratory Distress Syndrome (ARDS), sepsis, or septic shock, or other complications [12, 15].

Our study was approved by the Medical and Health Research Ethics Committee of the Faculty of Medicine, Public Health and Nursing, Universitas Gadjah Mada/Dr. Sardjito Hospital (KE/FK/0563/EC/2020). All participants or guardians signed a written informed consent for participating in this study.

## Results

### Phylogenetic analysis

**Fig. 1:**
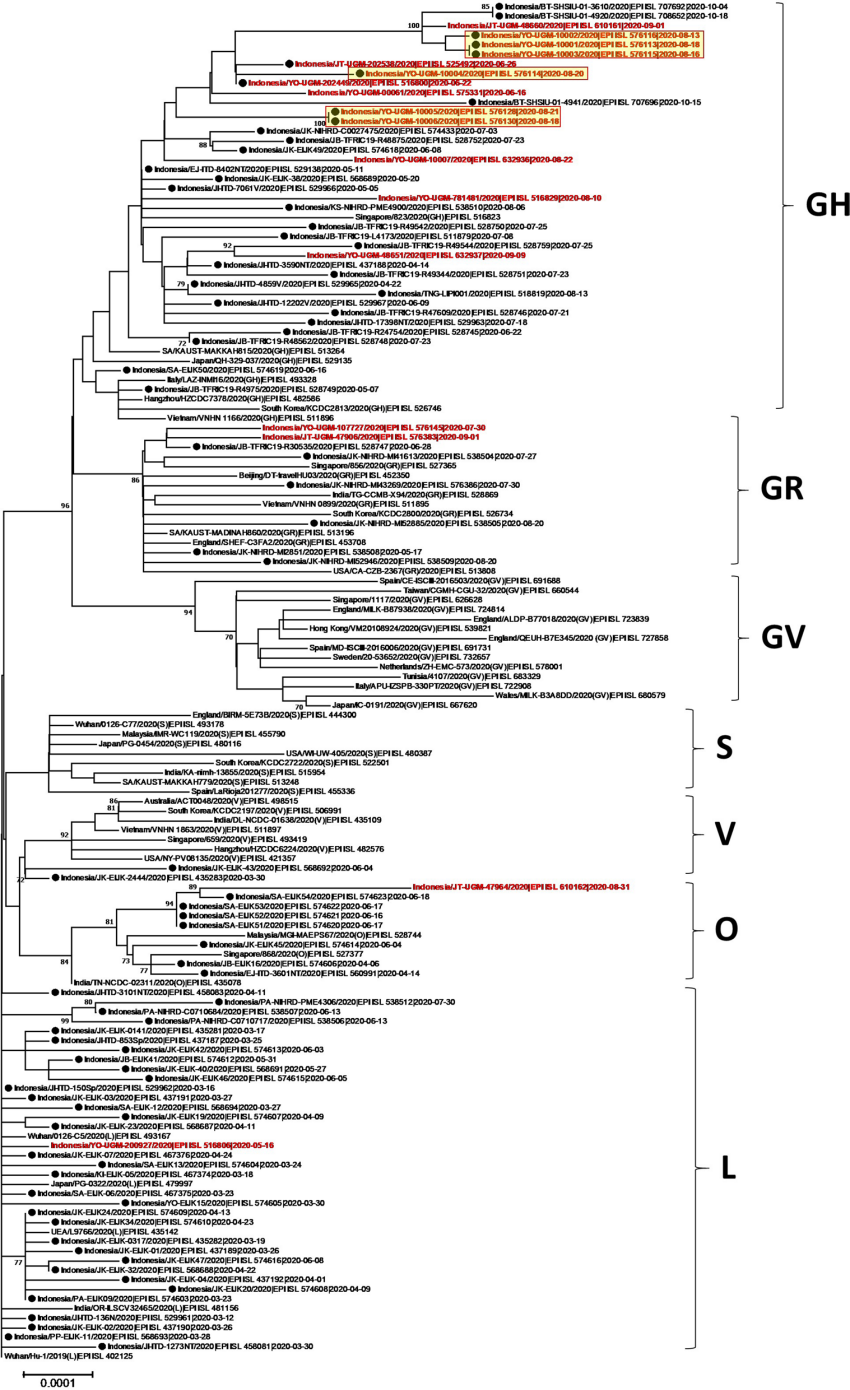
Phylogenetic analysis of SARS-CoV-2 genomes from Indonesia and different countries. A phylogenetic tree was constructed from 29.409 nt length of the open reading frame (ORF) of 142 SARS-CoV-2 virus sequences using Neighbor Joining statistical method with 2,000 bootstrap replications. The evolutionary distances were computed using the Kimura 2-parameter method and the rate variation among sites was modelled with a gamma distribution (estimated α = 0.14566). SARS-CoV-2 virus sequences from Indonesia (N = 89) followed by the date of collection are indicated in closed circles, while viruses for the study are indicated in red and viruses from the family clusters are color-shaded in yellow. The tree is rooted to Wuhan/Hu-1/2019 with the bootstrap percentage values less than 70% hidden and it is drawn to scale (0.0001) with branch lengths measured in the number of substitutions per site

Phylogenetic analysis revealed that thirteen virus samples were situated within clade GH (GISAID classification), while two viruses were grouped with other viruses which belonged to clade GR, and one virus each that belonged to clade O and clade L (Fig. 1). Three viruses from family cluster case-1 (YO-UGM-10001|EPI_ISL_576113, YO-UGM-10002|EPI_ISL_576114, and YO-UGM-10003|EPI_ISL_576115) formed a single group within clade GH, whereas viruses from family cluster-2 (YO-UGM-1004|EPI_ISL_576116, YO-UGM-1005|EPI_ISL_576128, and YO-UGM-1006|EPI_ISL_576130,) were separated into two different nodes within clade GH (Fig. 1, top-right).

### Molecular analysis

Ninety-four SNPs were detected throughout the ORP of the SARS-CoV-2 virus samples with 60% (54/94) of the nucleic acid changes resulting in amino acid substitutions (*missense* mutations) (Table 1, detailed in Additional file 1: Table S1). The types of nucleic acid base changes were more often detected as transitions (70%) compared to transversions (30%). Higher entropy values were observed more from nucleic acids that carried more frequent base changes; however, nucleic acid changes that caused missense mutation could have lower entropy values than those that resulted in synonymous mutation.

**Table 1:** Nucleic acid and amino acid mutations observed in seventeen SARS-CoV-2 virus genomes collected from Yogyakarta and Central Java provinces between June and September 2020

No.	NA position*	Gene/ Region	Number of SNP	NA change (SNP)	Type of base change	Entropy values	Frequency	Type of mutation	AA changes (position)^#^
1	1154	NSP2	7	**C → T**	*Transition*	0.21456	2	*Missense*	**A205V**
2	1280			**C → T**	*Transition*	0.34883	2	*Missense*	**V247A**
3	1307			**C → T**	*Transition*	0.45056	3	*Missense*	**T256I**
4	1501			**C → A**	*Transversion*	0.34883	2	*Missense*	**Q321K**
5	1845			**C → T**	*Transition*	0.21456	1	*Synonymous*	N435N
6	1998			C → T	*Transition*	0.21456	1	*Synonymous*	T486T
7	2247			A → G	*Transition*	0.21456	1	*Synonymous*	R569R
8	2772	NSP3	13	C → T	*Transition*	0.45056	15	*Synonymous*	F106F
9	3264			T → C	*Transition*	0.21456	1	*Synonymous*	D270D
10	3350			T → C	*Transition*	0.21456	1	*Synonymous*	V299A
11	3819			C → T	*Transition*	0.52971	4	*Synonymous*	G443G
12	4489			**C → T**	*Transition*	0.21456	1	*Missense*	**P679S**
13	4919			**C → T**	*Transition*	0.66825	13	*Missense*	**P822L**
14	5519			**C → T**	*Transition*	0.21456	1	*Missense*	**T1022I**
15	5541			C → T	*Transition*	0.34883	2	*Synonymous*	C1029C
16	5990			**C → T**	*Transition*	0.21456	1	*Missense*	**A1179V**
17	6047			**C → A**	*Transversion*	0.21456	1	*Missense*	**T1198K**
18	6515			**T → G**	*Transversion*	0.21456	1	*Missense*	**F1354C**
19	7374			C → T	*Transition*	0.21456	1	*Synonymous*	F1640F
20	7448			**C → T**	*Transition*	0.21456	1	*Missense*	**P1665L**
21	8981	NSP4	1	**C → T**	*Transition*	0.21456	1	*Missense*	**A231V**
22	9824	NSP5	4	**A → G**	*Transition*	0.52971	4	*Missense*	**K12R**
23	9936			**G → T**	*Transversion*	0.21456	1	*Missense*	**M49I**
24	10242			C → T	*Transition*	0.68696	8	*Synonymous*	N151N
25	10339			**C → T**	*Transition*	0.21456	1	*Missense*	**P184S**
26	10818	NSP6	1	**G → T**	*Transversion*	0.21456	1	*Missense*	**L37F**
27	11887	NSP8	2	**G → A**	*Transition*	0.21456	1	*Missense*	**A21T**
28	12174			C → T	*Transition*	0.21456	1	*Synonymous*	P116P
29	12544	NSP9	1	**C → T**	*Transition*	0.21456	1	*Missense*	**L42F**
30	13465	NSP12 (RdRp)	12	**C → T**	*Transition*	0.21456	1	*Missense*	**A97V**
31	13790			G → T	*Transversion*	0.21456	1	*Synonymous*	L205L
32	13855			**C → T**	*Transition*	0.21456	1	*Missense*	**P227L**
33	13918			**C → T**	*Transition*	0.52971	3	*Missense*	**T248I**
34	14027			C → T	*Transition*	0.21456	1	*Synonymous*	D284D
35	14143			**C → T**	*Transition*	0.45056	15	*Missense*	**P323L**
36	14429			C → T	*Transition*	0.21456	1	*Synonymous*	D418D
37	15141			**G → T**	*Transversion*	0.21456	1	*Missense*	**A656S**
38	15278			G → T	*Transversion*	0.21456	1	*Synonymous*	T701T
39	15500			A → G	*Transition*	0.21456	1	*Synonymous*	L775L
40	15849			**C → T**	*Transition*	0.34883	2	*Missense*	**H892Y**
41	15891			**A → G**	*Transition*	0.21456	1	*Missense*	**M906V**
42	16130	NSP13	8	A → T	*Transversion*	0.45056	3	*Synonymous*	P53P
43	16196			C → T	*Transition*	0.21456	1	*Synonymous*	H75H
44	16351			**C → T**	*Transition*	0.21456	1	*Missense*	**T127I**
45	16382			G → T	*Transversion*	0.52971	4	*Synonymous*	T137T
46	16429			**C → T**	*Transition*	0.21456	1	*Missense*	**T153I**
47	16476			**G → T**	*Transversion*	0.21456	1	*Missense*	**V169F**
48	16745			C → T	*Transition*	0.21456	1	*Synonymous*	I258I
49	17699			**G → T**	*Transversion*	0.21456	1	*Missense*	**M576I**
50	18382	NSP14	4	**C → T**	*Transition*	0.21456	1	*Missense*	**P203L**
51	18479			C → T	*Transition*	0.68696	8	*Synonymous*	Y235Y
52	18612			C → T	*Transition*	0.59084	14	*Synonymous*	L280L
53	18737			A → G	*Transition*	0.21456	1	*Synonymous*	L321L
54	19859	NSP15	2	T → C	*Transition*	0.21456	1	*Synonymous*	I168I
55	20364			**C → T**	*Transition*	0.21456	1	*Missense*	**H337Y**
56	20843	NSP16	2	C → T	*Transition*	0.21456	1	*Synonymous*	F150F
57	21058			**A → G**	*Transition*	0.21456	2	*Missense*	**Y222C**
58	21310	Spike (S)	12	**C → T**	*Transition*	0.34883	2	*Missense*	**L5F**
59	21387			T → C	*Transition*	0.21456	1	*Synonymous*	N30N
60	21477			C → T	*Transition*	0.52971	4	*Synonymous*	S60S
61	21483			T → C	*Transition*	0.21456	1	*Synonymous*	V62V
62	21544			**G → C**	*Transversion*	0.21456	1	*Missense*	**V83L**
63	21935			**T → C**	*Transition*	0.52971	4	*Missense*	**V213A**
64	22069			**T → C**	*Transition*	0.21456	1	*Missense*	**W258R**
65	23138			**A → G**	*Transition*	0.34883	16	*Missense*	**D614G**
66	23328			**G → T**	*Transversion*	0.21456	1	*Missense*	**Q677H**
67	23664			C → T	*Transition*	0.21456	1	*Synonymous*	Y789Y
68	23729			**A → T**	*Transversion*	0.21456	1	*Missense*	**N811I**
69	23928			G → T	*Transversion*	0.21456	1	*Synonymous*	L877L
70	25288	NS3	6	**C → T**	*Transition*	0.21456	1	*Missense*	**A54V**
71	25298			**G → T**	*Transversion*	0.59084	14	*Missense*	**Q57H**
72	25349			C → T	*Transition*	0.34883	2	*Synonymous*	S74S
73	25422			**G → T**	*Transversion*	0.21456	1	*Missense*	**A99S**
74	25579			**C → T**	*Transition*	0.21456	1	*Missense*	**T151I**
75	25791			**G → T**	*Transversion*	0.21456	1	*Missense*	**D222Y**
76	26470	M	3	C → T	*Transition*	0.59084	14	*Synonymous*	Y71Y
77	26536			C → A	*Transversion*	0.34883	2	*Synonymous*	L93L
78	26602			A → G	*Transition*	0.66825	7	*Synonymous*	E115E
79	27345	NS7a	1	**C → T**	*Transition*	0.21456	1	*Missense*	**H73Y**
80	27808	NS8	1	G → A	*Transition*	0.21456	1	*Synonymous*	L60L
81	28046	N	14	**C → T**	*Transition*	0.21456	1	*Missense*	**P13L**
82	28363			**G → T**	*Transversion*	0.52971	4	*Missense*	**A119S**
83	28470			T → C	*Transition*	0.21456	1	*Synonymous*	N154N
84	28479			C → T	*Transition*	0.21456	1	*Synonymous*	I157I
85	28487			**A → G**	*Transition*	0.21456	1	*Missense*	**Q160R**
86	28586			**G → T**	*Transversion*	0.52971	4	*Missense*	**S193I**
87	28593			**A → T**	*Transversion*	0.21456	1	*Missense*	**R195S**
88	28603			**C → T**	*Transition*	0.21456	1	*Missense*	**P199S**
89	28616			**G → A**	*Transition*	0.34883	2	*Missense*	**R203K**
90	28617			**G → A**	*Transition*	0.34883	2		
91	28618			**G → C**	*Transversion*	0.34883	2	*Missense*	**G204R**
92	28710			**G → T**	*Transversion*	0.21456	1	*Missense*	**M234I**
93	28944			A → G	*Transition*	0.21456	1	*Synonymous*	S312S
94	29094			A → T	*Transversion*	0.34883	2	*Synonymous*	T362T

Bold indicates non-synonymous substitutions

^*^Nucleic acid numbering starting from ORF1ab start codon (ATG)

^#^Amino acid numbering starting from start codon of each gene

The majority of the virus samples (16/17) possessed D614G substitution on spike protein and 56% of these (9/16) showed other amino acid substitutions on this protein, including L5F, V83L, V213A, W258R, Q677H, and N811I. Second amino acid mutations that were frequently detected were P232L substitution on NSP12 (RdRp) protein (15x), followed by Q57H substitution on NS3 (14x) and P822L substitution on NSP3 protein (13x). Furthermore, various amino acid mutations were also found in the other proteins of virus samples, including on **NSP2** (A205V, V247A, T256I, Q321K), **NSP3** (P679S, T1022I, A1179V, T1198K, F1354C, P1665L), **NSP4** (A231V), **NSP5** (K12R, M49I, P184S), NSP6 (L37F), **NSP8** (A21T), NSP9 (L42F), **NSP12/RdRp** (A97V, P227L, T248I, A656S, H892Y, M906V), **NSP13** (T127I, T153I, V169F, M576I, P203L), **NSP15** (H337Y), **NSP16** (Y222C), **NS3** (A54V, A99S, T151I, D222Y), **NS7a** (H73Y), and **N** (P13L, A119S, Q160R, S193I, R195S, P199S, R203K, G204R, M234I).

### COVID-19’s severity and spike protein mutations of COVID-19 samples

Based on the case definition of COVID-19 severity developed for this study, 3 of 17 virus samples (17.6%) were collected each from asymptomatic cases (people) and critical cases, 5 virus samples (29.4%) from mild cases, and 6 virus samples (35.3%) from moderate cases (Table 2). Two of the patients with critical stages eventually died. A range of Ct values was found amongst different stages of severity, nevertheless all the virus samples with D614G mutations, except one (YO-UGM-10004/2020|EPI_ISL_576116), showed lower Ct values (clade GH, GR, and O, Ct range 16.9–24.7) than those with no mutation in this position (clade L, Ct 27.9). Dual mutations of V213A and D614G on spike protein were detected in four patients, and two of these eventually died after a period of hospitalization.

**Table 2:** Severity and genetic data associated with SARS-CoV-2 viruses collected from seventeen COVID-19 patients in Yogyakarta and Central Java provinces, Indonesia from June–September 2020

Patient no.	Sex	Age (yo)	COVID-19 severity	C_T_ value	Virus name (GISAID Accession ID)	Average coverage	Collection date	Lineage (GISAID clade)	Spike mutations
1	Male	30	Mild	27.9	hCoV19/Indonesia/YO-UGM-200927/2020 (EPI_ISL_516806)	102x	16/05/2020	L	-
2	Female	49	Asymptomatic	22.31	hCoV-19/Indonesia/YO-UGM-00061/2020 (EPI_ISL_575331)	4655x	16/06/2020	GH	D614G
3	Male	77	Moderate	19.7	hCoV19/Indonesia/YO-UGM-202449/2020 (EPI_ISL_516800)	22088x	22/06/2020	GH	D614G
4	Female	55	Moderate	24.7	hCov19/Indonesia/JT-UGM-202538/2020 (EPI_ISL_525492)	347x	26/06/2020	GH	D614G
5	Male	28	Asymptomatic	21.05	hCoV-19/Indonesia/YO-UGM-107727/2020 (EPI_ISL_576145)	104x	30/07/2020	GR	D614G
6	Female	83	Moderate	16.9	hCoV19/Indonesia/YO-UGM-781481/2020 (EPI_ISL_516829)	3748x	10/08/2020	GH	D614G
7	Male	28	Critical	20	hCoV-19/Indonesia/YO-UGM-10002/2020 (EPI_ISL_576114)*	13x	13/08/2020	GH	V213A, D614G
8	Male	58	Critical (Died)	23	hCoV-19/Indonesia/YO-UGM-10003/2020 (EPI_ISL_576115)*	505x	16/08/2020	GH	V213A, D614G
9	Male	88	Critical (Died)	18.1	hCoV-19/Indonesia/YO-UGM-10001/2020 (EPI_ISL_576113)*	42888x	18/08/2020	GH	V213A, D614G
10	Male	8	Moderate	19.5	hCoV-19/Indonesia/YO-UGM-10006/2020 (EPI_ISL_576130)**	26x	18/08/2020	GH	L5F, D614G
11	Male	35	Mild	32	hCoV-19/Indonesia/YO-UGM-10004/2020 (EPI_ISL_576116)**	45x	20/08/2020	GH	D614G
12	Female	33	Mild	22	hCoV-19/Indonesia/YO-UGM-10005/2020 (EPI_ISL_576128)**	106x	21/08/2020	GH	L5F, D614G
13	Female	52	Asymptomatic	18	hCoV-19/Indonesia/YO-UGM-10007/2020 (EPI_ISL_632936)	2207x	22/08/2020	GH	D614G, N811I
14	Male	48	Moderate	19.64	hCoV-19/Indonesia/JT-UGM-47964/2020 (EPI_ISL_610162)	6291x	31/08/2020	O	W258R, D614G
15	Male	36	Mild	17.53	hCoV-19/Indonesia/JT-UGM-47906/2020 (EPI_ISL_576383)	653x	01/09/2020	GR	D614G
16	Female	64	Moderate	19.44	hCoV-19/Indonesia/JT-UGM-48660/2020 (EPI_ISL_610161)	22x	01/09/2020	GH	V213A, D614G
17	Male	41	Mild	21.24	hCoV-19/Indonesia/YO-UGM-48651/2020 (EPI_ISL_632937)	2867x	09/09/2020	GH	V83L, D614G, Q677H

Virus samples collected from family cluster-1 *) and from family cluster-2 **)

CT, cycle threshold; Ref. sequence: hCoV-19/Wuhan/Hu-1/2019 (NC_045512.2)

### Disease outcomes of COVID-19’s family clusters

**Fig. 2:**
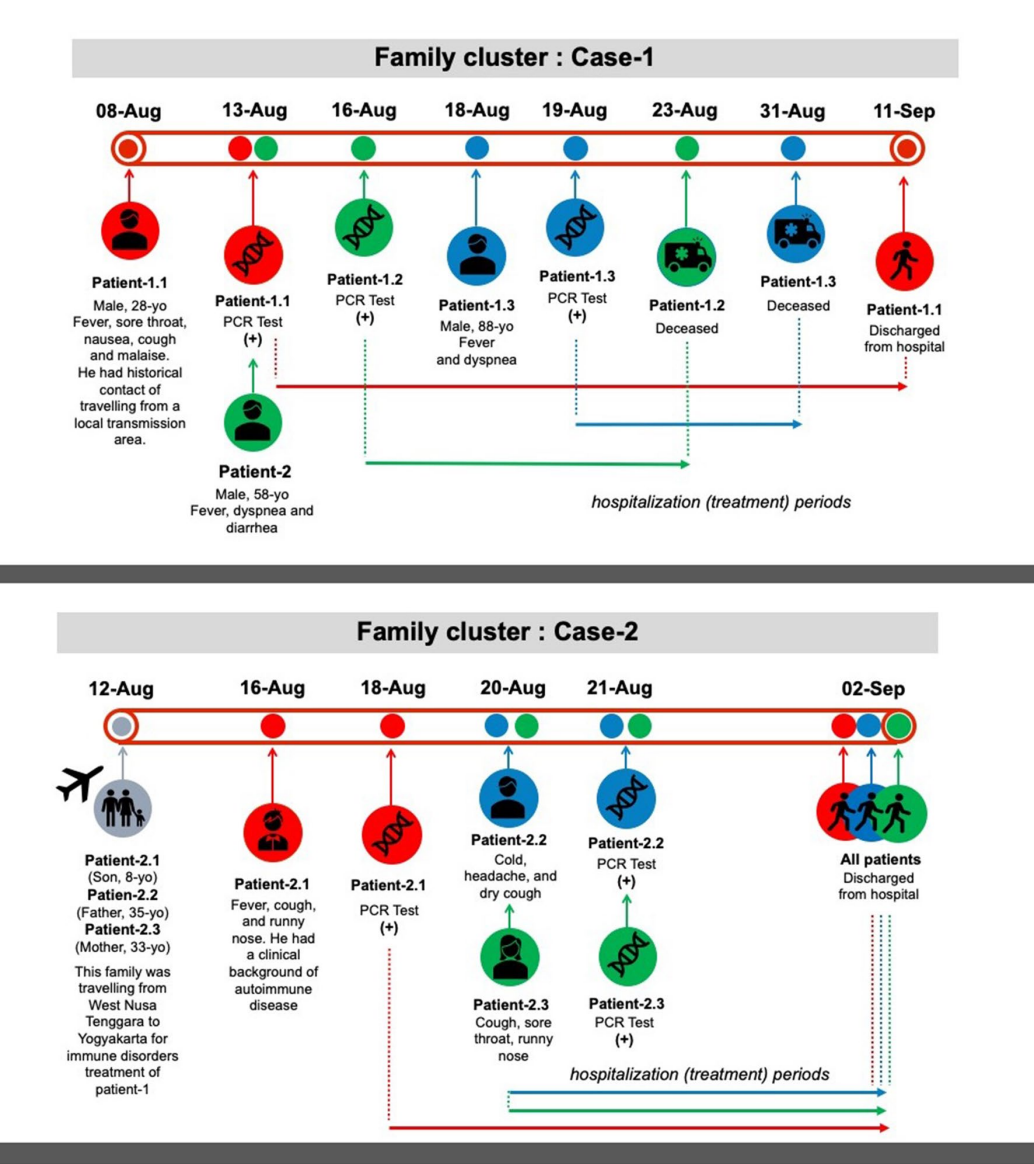
Timeline of COVID-19 symptoms and diagnosis from family clusters in Yogyakarta and Central Java

The epidemiological and clinical data of COVID-19’s family clusters, including clinical symptoms, date of first symptoms appeared, diagnostic results, abnormal findings, comorbidity background are provided by timeline and tabulation in Fig. 2 and Table 3, respectively.

**Table 3:** Characteristics of patients with COVID-19 from family cluster cases in Yogyakarta and Central Java

Patient no.	Sex	Age (yo)	COVID-19 severity	Symptoms	Date of first symptoms appeared	Abnormal findings	Comorbidity	Virus name (GISAID Accession ID)
1.1	Male	28	Critical	Fever, sore throat, nausea, cough, malaise	08/08/2020	CXR: typical COVID-19 bilateral pneumonia NLR 12.2 ARDS PaO2/FIO2 260	Obesity	hCoV-19/Indonesia/YO-UGM-10002/2020 (EPI_ISL_576114)
1.2	Male	58	Critical (Died)	Fever, dyspnea, diarrhea	13/08/2020	Crackles in both lungs NLR 8.2 5th days ARDS PaO2/FIO2 68 (intubated)	DM, Hypertension and obesity	hCoV-19/Indonesia/YO-UGM-10003/2020 (EPI_ISL_576115)
1.3	Male	88	Critical (Died)	Fever, dyspnea	19/08/2020	Crackles in both lungs CXR: bilateral pneumonia and cardiomegaly Electrolyte imbalance, PaO2/FIO2 123.3 (worsening ARDS) Intubated on 7th day	Type 2 DM, Geriatric syndrome, History of infarction stroke	hCoV-19/Indonesia/YO-UGM-10001/2020 (EPI_ISL_576113)
2.1	Male	8	Moderate	Fever, cough, runny nose	16/08/2020	CXR: bilateral paracardial infiltrate	Autoimmune disorders (Henoch-Schönlein purpura)	hCoV-19/Indonesia/YO-UGM-10006/2020 (EPI_ISL_576130)
2.2	Male	35	Mild	Cold, headache, dry cough	20/08/2020	Eosinophilia (6.4%) pH (7.45), PaO2 (83.2), PaCO2 (39.2), SaO2 (96.8), PaO2/FiO2 (416)	Hypertension	hCoV-19/Indonesia/YO-UGM-10004/2020 (EPI_ISL_576116)
2.3	Female	33	Mild	Cough, sore throat, runny nose	20/08/2020	No abnormality	None	hCoV-19/Indonesia/YO-UGM-10005/2020 (EPI_ISL_576128)

*CXR* chest X-ray, *ARDS* acute respiratory distress syndrome, *NLR* neutrophil to lymphocyte ratio, *DM* diabetes mellitus

In family cluster-1, all three patients showed critical COVID-19 and eventually, two died (YO-UGM-10001|EPI_ISL_576113 and YO-UGM-10003|EPI_ISL_576115) and one survived (YO-UGM-10002|EPI_ISL_576114). The disease began from patient-1.1, a 28-year-old male, who had a history of traveling from the local COVID-19 transmission area. He complained of fever, sore throat, cough and malaise on August 8th, 2020, and was tested for PCR three days afterward with the result of COVID-19 positive. His father (patient-1.2, 58-yo), who was living in the same house, showed fever, dyspnea and diarrhea on August 13th, then was followed by his grandfather (patient-1.3, 88-yo) who showed fever and dyspnea on August 18th. The PCR tests for both patients showed positive for COVID-19. All patients developed severe disease outcomes including bilateral pneumonia, cardiomegaly and ARDS. Several comorbidities were recorded from patient-1.1 (obesity), patient-1.2 (diabetes mellitus, and obesity), and patient-1.3 (type 2 diabetes mellitus, geriatric syndrome, history of infarction stroke). Patient-1.1 was uneventfully discharged from the hospital on day 29 of hospitalization, but sadly, patient-1.2 and patient-1.3 passed away in the hospital after 7 and 12 days of hospitalization, respectively.

Family cluster-2 involved three patients which were comprised of a son, 8-yo (patient-2.1), father, 35-yo (patient-2.2) and mother, 33-yo, with the following virus samples: YO-UGM-10006 (EPI_ISL_576130), YO-UGM-10004/2020 (EPI_ISL_576114), and YO-UGM-10005/2020 (EPI_ISL_576128), respectively. Prior to the index case, this family travelled from West Nusa Tenggara to Yogyakarta on August 2nd, 2020, in order to obtain medical treatment for patient-2.1 who had an autoimmune disorder in a hospital in Yogyakarta. Patient-2.1 had firstly exhibited symptoms of fever, cough, and runny nose on August 11th, 2020 and he was diagnosed COVID-19 positive on August 18th. His parents showed clinical signs of cold, headache and dry cough (patient-2.2) and cough, sore throat, and runny nose (patient-2.3) at the same day on August 20th and the PCR results of both patients were positive on August 21st. Patient-2.1 developed moderate severity with bilateral paracardial infiltrate, whereas patient-2.2 and patient-2.3 developed mild disease without any abnormalities in their chest X-rays and other laboratory findings, except eosinophilia (6.4%), increased levels in pH of the blood (7.45), PaO_2_ (83.2), PaCO_2_ (39.2) with SaO_2_ 96.8% and PaO_2_/FiO_2_ value was 416 from the arterial blood glass analysis of patient-2.2. All three patients uneventfully recovered and were discharged from the hospital on September 2nd, 2020.

### Molecular characterizations of virus samples collected from family clusters

**Fig. 3:**
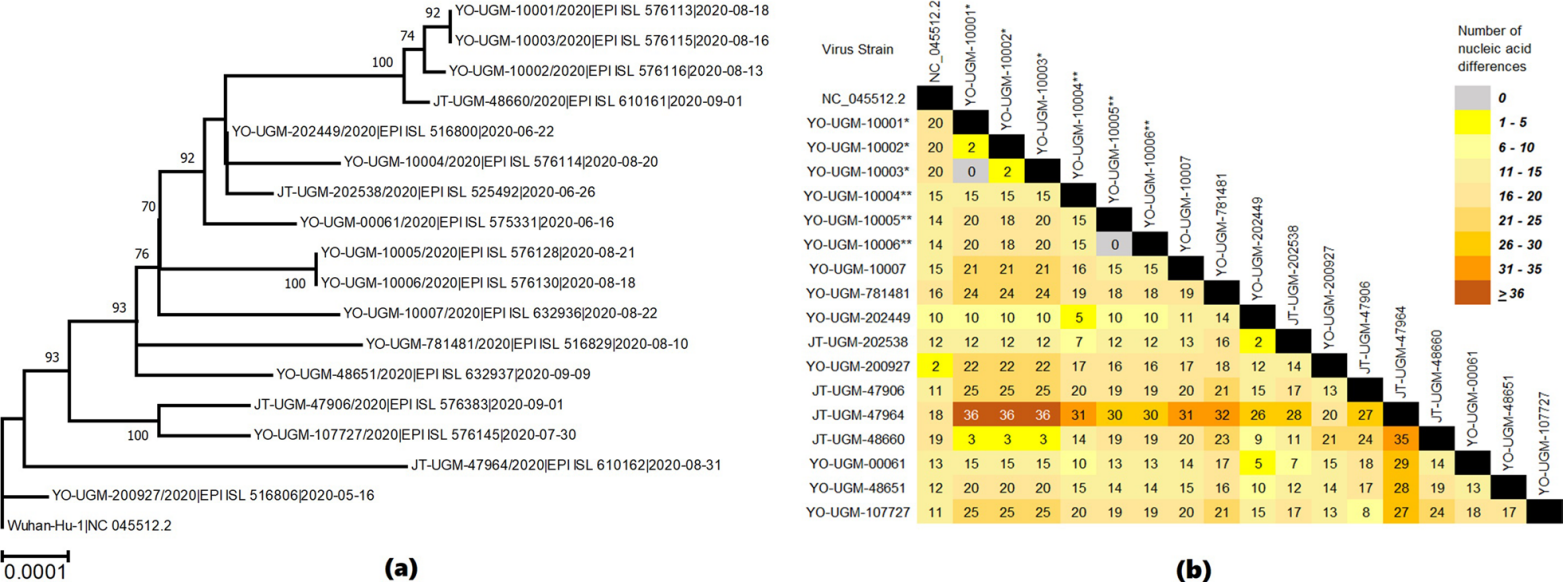
Phylogenetic analysis and nucleic acid differences of family clusters’ virus sequences compared to the other SARS-CoV-2 virus sequences from Yogyakarta and Central Java. **a** The tree was constructed from 29.409 nt length of the open reading frame (ORF) of 142 SARS-CoV-2 virus sequences using Neighbor Joining statistical method and computed using the Kimura 2-parameter method with 2,000 bootstrap replications. The tree is rooted to Wuhan/Hu-1/2019 (NC_045512.2) with the bootstrap percentage values less than 70% hidden and it is drawn to scale (0.0001) with branch lengths measured in the number of substitutions per site. **b** The number of base differences per sequence from between sequences are shown. Codon positions included were 1st + 2nd + 3rd + Noncoding. All ambiguous positions were removed for each sequence pair. There was a total of 29,409 positions in the final dataset. Family clusters are indicated in asterisks: Case-1 (*) and Case-2 (**)

Phylogenetic analysis revealed that the three viruses from family cluster-1 were grouped together from a single node. A matrix of nucleic acid difference showed that YO-UGM-10001|EPI_ISL_576113 and YO-UGM-10003|EPI_ISL_576115 were identical on their ORF (nucleic acid and protein levels) and both virus strains had differences of 2 nucleic acids and 1 amino acid in the NSP2 protein which correspond with V247A substitution in YO-UGM-10001|EPI_ISL_576113 and YO-UGM-10003|EPI_ISL_576115 and T256I substitution in YO-UGM-10002|EPI_ISL_576114, respectively (Table 4). Other unique mutations in the other viral proteins were detected in these three virus strains which were not shown in the other study viruses, including V213A (Spike), K12R (NSP5), T248I (NSP12/RdRp), A119S and S193I (N). The virus samples from family cluster-2 were separated in different nodes in the phylogenetic tree (Fig. 3). The tree and the matrix sequence showed that YO-UGM-10005/2020|EPI_ISL_576128 and YO-UGM-10006|EPI_ISL_576130 were genetically identical. Both virus strains had 15 nucleic acid differences compared to YO-UGM-10004|EPI_ISL_576114 which resulted in amino acid variations detected in several viral proteins (Table 4).

**Table 4:** Amino acid mutations detected in SARS-CoV-2 viruses collected from two family cluster cases in Yogyakarta and Central Java provinces

Virus (Accession No.)	NSP2			NSP3		NSP4	NSP5	NSP12 (RdRp)		
Amino acid position in each gene	247	256	321	299	822	231	12	248	323	892
*Wuhan/19 (NC_045512.2)*	*V*	*T*	*Q*	*V*	*P*	*A*	*K*	*T*	*P*	*H*
YO-UGM-10001 (EPI_ISL_576113)*	**A**	T	Q	V	**L**	A	**R**	**I**	**L**	H
YO-UGM-10002 (EPI_ISL_576114)*	V	**I**	Q	V	**L**	A	**R**	**I**	**L**	H
YO-UGM-10003 (EPI_ISL_576115)*	**A**	T	Q	V	**L**	A	**R**	**I**	**L**	H
YO-UGM-10004 (EPI_ISL_576116)**	V	T	Q	**A**	**L**	**V**	K	T	**L**	H
YO-UGM-10005 (EPI_ISL_576128)**	V	**I**	**K**	V	**L**	A	K	T	**L**	**Y**
YO-UGM-10006 (EPI_ISL_576130)**	V	**I**	**K**	V	**L**	A	K	T	**L**	**Y**
YO-UGM-10007 (EPI_ISL_632936)	V	T	Q	V	**L**	A	K	T	**L**	H
YO-UGM-781481 (EPI_ISL_516829)	V	T	Q	V	P	A	K	T	**L**	H
YO-UGM-202449 (EPI_ISL_516800)	V	T	Q	V	**L**	A	K	T	**L**	H
JT-UGM-202538 (EPI_ISL_525492)	V	T	Q	V	**L**	A	K	T	**L**	H
YO-UGM-200927 (EPI_ISL_516806)	V	T	Q	V	P	A	K	T	P	H
JT-UGM-47906 (EPI_ISL_576383)	V	T	Q	V	P	A	K	T	**L**	H
JT-UGM-47964 (EPI_ISL_610162)	V	T	Q	V	P	A	K	T	P	H
JT-UGM-48660 (EPI_ISL_610161)	V	T	Q	V	**L**	A	**R**	T	**L**	H
YO-UGM-00061 (EPI_ISL_575331)	V	T	Q	V	**L**	A	K	T	**L**	H
YO-UGM-48651 (EPI_ISL_632937)	V	T	Q	V	P	A	K	T	**L**	H
YO-UGM-107727 (EPI_ISL_576145)	V	T	Q	V	P	A	K	T	**L**	H

Virus (Accession No.)	NSP13	NSP14	Spike			NS3	N			
Amino acid position in each gene			5	213	614	57	119	193		
*Wuhan/19 (NC_045512.2)*	*T*	*P*	*L*	*V*	*D*	*Q*	*A*	*S*		
YO-UGM-10001 (EPI_ISL_576113)*	T	P	L	**A**	**G**	**H**	**S**	**I**		
YO-UGM-10002 (EPI_ISL_576114)*	T	P	L	**A**	**G**	**H**	**S**	**I**		
YO-UGM-10003 (EPI_ISL_576115)*	T	P	L	**A**	**G**	**H**	**S**	**I**		
YO-UGM-10004 (EPI_ISL_576116)**	**I**	**L**	L	V	**G**	**H**	A	S		
YO-UGM-10005 (EPI_ISL_576128)**	T	P	**F**	V	**G**	**H**	A	S		
YO-UGM-10006 (EPI_ISL_576130)**	T	P	**F**	V	**G**	**H**	A	S		
YO-UGM-10007 (EPI_ISL_632936)	T	P	L	V	**G**	**H**	A	S		
YO-UGM-781481 (EPI_ISL_516829)	T	P	L	V	**G**	**H**	A	S		
YO-UGM-202449 (EPI_ISL_516800)	T	P	L	V	**G**	**H**	A	S		
JT-UGM-202538 (EPI_ISL_525492)	T	P	L	V	**G**	**H**	A	S		
YO-UGM-200927 (EPI_ISL_516806)	T	P	L	V	D	Q	A	S		
JT-UGM-47906 (EPI_ISL_576383)	T	P	L	V	**G**	Q	A	S		
JT-UGM-47964 (EPI_ISL_610162)	T	P	L	V	**G**	Q	A	S		
JT-UGM-48660 (EPI_ISL_610161)	T	P	L	**A**	**G**	**H**	**S**	**I**		
YO-UGM-00061 (EPI_ISL_575331)	T	P	L	V	**G**	**H**	A	S		
YO-UGM-48651 (EPI_ISL_632937)	T	P	L	V	**G**	**H**	A	S		
YO-UGM-107727 (EPI_ISL_576145)	T	P	L	V	**G**	Q	A	S		

Family clusters are indicated in asterisks: Case-1 (*) and Case-2 (**). In family cluster-1 and 2, variations in the amino acids of the NSP2 protein (V247A and T256I, and T256I and Q321K, respectively) were noted, but not in others. The virus samples isolated from fatal disease outcomes of family cluster-1 (YO-UGM-10001|EPI_ISL_576113 and YO-UGM-10003|EPI_ISL_576115) carried V247A mutation in the NSP2 protein, while those from the recovered patient (YO-UGM-10002|EPI_ISL_576114) did not. Bold, amino acids were different from their reference (Wuhan/19 [NC_045512.2])

## Discussion

Our study provides evidence of SARS-CoV-2 transmission within families, in which the same mutation of the spike protein in each family cluster was identified. It is important to understand the transmission routes of SARS-CoV-2 to prevent and control its spreading [4]. Families have been reported as the most dominant infection cluster of COVID-19 [16]. Family clusters have a higher risk of cross-infection because of frequent and close contact among each family member [4]. Our study also documented that although all family members showed the same multiple S protein mutations, however, they revealed different outcomes. While multiple S protein mutations, i.e. B.1.1.7 variant, have been associated with the severity of COVID-19 [17, 18], this is not the case for our patients. Our samples did not consist of B.1.1.7 variant. In addition, several prognostic factors have been associated with increased risk of severity and mortality of COVID-19, including increasing age, obesity, and comorbidities such as hypertension, diabetes and cerebrovascular disease [15]. Our patients who eventually died (YO-UGM-10001|EPI_ISL_576113 and YO-UGM-10003|EPI_ISL_576115) have more prognostic factors than the patient who survived (YO-UGM-10002|EPI_ISL_576114) (Table 3). Besides the SARS-CoV-2 variants and prognostic factors, a recent GWAS identified rs11385942 at locus 3p21.31 and rs657152 at locus 9q34.2 as a genetic risk factor for severe COVID-19 [19]. Further study is necessary to confirm whether these polymorphisms might be as susceptible factors in our patients.

Double mutations of V213A and D614G on spike protein were detected in four patients, but three of them (75%) developed severe diseases causing critical conditions and two (50%) with fatal outcome. Another interesting finding was documented from family cluster-1, in which all the virus samples in this family cluster belong to the same clade GH, but two patients died and one survived (Table 2). All samples from family cluster-1 revealed another spike protein mutation, V213A, besides D614G. However, virus samples isolated from fatal disease outcomes carried V247A mutation in the NSP2 protein, while those from the recovered patient did not. In conjunction with D614G mutation, substitution of valine (V) to alanine (A) in position 247 and 213 of NSP2 and spike protein, respectively, were detected in the patients with fatal disease outcomes. While both V and A, as well as G are in the non-polar hydrophobic amino acid group and no evidence shows that the double mutations of V213A and D614G affect the severity and lethality of COVID-19 patients, further investigations are necessary to determine whether these dual mutations (V213A and D614G in spike protein) or even triple mutations (V213A and D614G in spike protein and V47A in NS2) associated with increased risk of mortality in COVID-19 patients. Moreover, due to limited number of sample size in this study, it is very difficult to associate between the number of mutations on the spike protein or other proteins or SNPs and severity of COVID-19.

Phylogenetic analysis revealed that three viruses from family cluster-1 formed a monophyletic group. The epidemiological and genetic data indicated that local transmission occurred in family cluster-1 in which patient-1.1 (YO-UGM-10002|EPI_ISL_576114) was initially infected and then transmitted the virus to patient-1.2 (YO-UGM-10003|EPI_ISL_576115) and patient-1.3 (YO-UGM-10001|EPI_ISL_576113). Interestingly, the virus that infected patient 2.2 in family cluster-2 was genetically different from that which infected both two counterparts: patient 2.1 (YO-UGM-10006/2020 (EPI_ISL_576130) and patient 2.3 (YO-UGM-10005/2020 (EPI_ISL_576128). These viruses formed a polyphyletic group indicating there is the possibility of different sources of infection (two convergent descendants, but not their common ancestors).

Recently, more than 50% of the viral genome sequences in the UK were reported to have a new single phylogenetic cluster, i.e. B.1.1.7 variant (multiple spike protein mutations: deletion 69–70, deletion 144, N501Y, A570D, D614G, P681H, T716I, S982A, D1118H) [9]. These new variants have been associated with a higher transmissibility of SARS-CoV-2 up to 70% [9]. Until the submission date of April 2021 in GISAID, these variants were also detected in Asia, including Indonesia [11]. Interestingly, we detected other spike protein mutations in our collected virus strains, including those from the family clusters, i.e. L5F, V213A, W258R, Q677H, and K811I. Noteworthy, the V213A variant was identified in all patients from family cluster-1. V213A was detected in 4/17 (23.5%) of our samples. This variant is only found in only 0.01% of samples in four countries, including Indonesia [11]. Whether this variant is due to a founder effect needs further study.

Currently, besides the D614G variant, several mutations within the receptor binding domains (RBD) of the S protein have attracted most scientists’ attention due to their increased frequency in certain countries, including S477N (Australia and some Central European), N439K (UK and European), and N501Y (part of the new UK variant B.1.1.7, the new South Africa variant 501.V2 and the new Brazil variant P.1) [11]. These variants might be associated with some potential advantages for these viruses. While the B.1.1.7 variant has been associated with COVID-19 clinical severity [17, 18], the 501.V2 and P.1 variants have not [20].

In addition, among eight clades in the GISAID classification, we only detected five clades, i.e. L, G, GH, GR, and O, in the SARS-CoV-2 samples from Indonesia and most of them (~ 60%) contained D614G. Globally, D614G has been detected in ~ 97% samples in 182 countries [11]. While a recent study showed that D614G mutation is significantly associated with the increase of SARS-CoV-2 infectivity, competitive fitness, and transmission in primary human airway epithelial cells and hamsters [21], it does not associate with the clinical severity of COVID-19 patients [22]. Moreover, it is difficult to assess the convergent evolution of D614G mutation in our samples since all samples were from Yogyakarta and Central Java and D614G has been already found in most samples (97%) from all over the world [11]. These findings were compatible with previous studies [22, 23]. The hypothesis of convergent evolution for D614G mutation is not supported by the sequence data since almost all 614G variants derived from the same ancestor [23]. Volz et al*.* [22] proposed a more complex selective landscape in the spike protein for the co-occurring variants between D614G and the neighbouring sites (615 and 613).

Phylogenetic analysis showed that the full-genome sequences of SARS-CoV-2 identified within these family clusters are identical, which strongly indicates a direct transmission within these families. Moreover, our study is also able to determine the virus clades of COVID-19 cases with unknown contact history with a confirmed COVID-19 case. Our findings support a previous suggestion regarding the importance of genomic epidemiology in filling the gaps of identifying SARS-CoV-2 infection sources [6]. Therefore, a full-genome surveillance of SARS-CoV-2 in Indonesia is essential to prevent further transmission of SARS-CoV-2 and to identify any established or new variant that might affect the SARS-CoV-2 transmission and severity.

Notably, our study only included a limited number of family clusters from Yogyakarta and Central Java, Indonesia. These limitations should be considered for interpretations of our findings.

## Conclusions

This is the first molecular epidemiology study associating the full-genome sequences of SARS-CoV-2 with the epidemiological and clinical data within family clusters. Phylogenetic analysis revealed that the three viruses from family cluster-1 formed a monophyletic group, whereas viruses from family cluster-2 formed a polyphyletic group indicating there is the possibility of different sources of infection. This study highlights how the same spike protein mutations among members of the same family might show different disease outcomes. Moreover, we also detected multiple spike protein mutations in our samples. Further studies are necessary to clarify the impact of these multiple spike protein mutations in the transmission and severity of SARS-CoV-2 infection, especially in Indonesia.

## Supplementary Information

**Additional file 1: Table S1.** Ninety-four SNPs were detected throughout the ORP of the SARS-CoV-2 virus samples with 60% (54/94) of the nucleic acid changes resulting in amino acid substitutions (missense mutations).

**Additional file 2: Table S2** The Acknowledgments Table for GISAID is report.

## Data Availability

All data generated or analyzed during this study are included in the submission. The sequence and metadata are shared through GISAID (www.gisaid.org).

## Abbreviation

SNPs: Single nucleotide polymorphisms
